# Peer creation and sharing of mnemonics in collaborative documents for pathology education: a pilot study

**DOI:** 10.1186/s12909-024-05743-1

**Published:** 2024-07-09

**Authors:** Bernd F. M. Romeike

**Affiliations:** Dean’s Office for Student Affairs, Medical Education, University Medical Center, Ernst-Heydemann-Strasse 8, D-18057 Rostock, Germany

**Keywords:** Collaborative documents, Digital literacy, Dynamic online learning, Instructional design, Medical education, Mnemonics, Pathology education, Peer creating, Peer sharing

## Abstract

**Background:**

Mnemonic techniques are memory aids that could help improve memory encoding, storage, and retrieval. Using the brain's natural propensity for pattern recognition and association, new information is associated with something familiar, such as an image, a structure, or a pattern. This should be particularly useful for learning complex medical information. Collaborative documents have the potential to revolutionize online learning because they could increase the creativity, productivity, and efficiency of learning. The purpose of this study was to investigate the feasibility of combining peer creation and sharing of mnemonics with collaborative online documents to improve pathology education.

**Methods:**

We carried out a prospective, quasi-experimental, pretest–posttest pilot study. The intervention group was trained to create and share mnemonics in collaborative documents for pathological cases, based on histopathological slides. The control group compared analog and digital microscopy.

**Results:**

Both groups consisted of 41 students and did not reveal demographic differences. Performance evaluations did not reveal significant differences between the groups' pretest and posttest scores. Our pilot study revealed several pitfalls, especially in instructional design, time on task, and digital literacy, that could have masked possible learning benefits.

**Conclusions:**

There is a gap in evidence-based research, both on mnemonics and on CD in pathology didactics. Even though, the combination of peer creation and sharing of mnemonics is very promising from a cognitive neurobiological standpoint, and collaborative documents have great potential to promote the digital transformation of medical education and increase cooperation, creativity, productivity, and efficiency of learning. However, the incorporation of such innovative techniques requires meticulous instructional design by teachers and additional time for students to become familiar with new learning methods and the application of new digital tools to promote also digital literacy. Future studies should also take into account validated high-stakes testing for more reliable pre-posttest results, a larger cohort of students, and anticipate technical difficulties regarding new digital tools.

**Supplementary Information:**

The online version contains supplementary material available at 10.1186/s12909-024-05743-1.

## Background

### Mnemonics in pathology education

For students in the medical field, the sheer volume of information in pathology can be overwhelming. The slightest tissue changes in histopathology can signify a wide spectrum of differential diagnoses. There are many patterns and medical terms that must be learned. Traditional pedagogy often fails to address the diverse learning styles of today's digital native students. This requires the exploration of new educational strategies to enhance learning outcomes.

Mnemonics have been used as a memory enhancement tool in pathology education [1–11]. Their value lies in condensing complex information into easily digestible and memorable fragments. Using cognitive shortcuts and associative memory, mnemonics transform challenging concepts into tangible and accessible pieces of knowledge. This involves fundamental linking of new information with existing long-term memory, usually with the help of mental images, which ultimately result in a memorable story. The most popular types of mnemonics in medical education are described in Table 1.

**Table 1:** Types of mnemonics, definitions and examples in pathology education

Mnemonic type	Definition	Example in pathology education
Abecedarius	An acrostic where the initial letters follow the alphabet sequentially	[4] The **ABC** of lead poisoning: **A**nemia, **B**asophilic stippling of red blood cells, **C**olic abdominal pain, **D**ementia, **E**ncephalopathy, **F**oot drop, and **G**ingival pigmentation
Acronym	A word formed from the initial letters of a term, each letter representing a word to remember	FISH representing fluorescence in situ hybridization
Acrostic	A sentence where the first letter of each word in a phrase or line stands for a word or idea to remember	[2] Remember types of necrosis with **L**ife **C**an **G**et **C**omplicated which stands for **L**iquefactive, **C**oagulation, **G**angrene, **C**aseous
Eponyms	A term in which a tissue or lesion is visualized or likened to the appearance of an everyday object, frequently food	[9] The most commonly used mnemonics in pathology are food eponyms: renal arteriosclerosis can be compared to onion skin
Loci method	A memory palace or a predefined list of specific physical locations (e.g. body parts), then imagery is used to store items at sites, items are retrieved, as you walk along sites	Create a body parts list from 1–12: 1 foot, 2 ankle, 3 knee, 4 hip, 5 belly, 6 chest, 7 neck, 8 chin, 9 mouth, 10 nose, 11 eyes, 12 ears; then imagine and place pathological conditions in correct order at specific locations; e.g. occurrence of certain tumors, mutations, use of medication
Rhyme system	Using rhyming words to aid memory	"Parkinson shakes, dopamine breaks"
Shape system	Using shapes or outlines to aid memory	The outline of a given histomorphological slide might remind you on something, e.g. a diamond
Similarities and differences	Grouping information according to similarities or differences	[2] The 3 Ms’ of cytological changes of herpes infection: **M**ultinucleation, **M**olding (nuclear), **M**argination of nuclear chromatin
Soundalikes and chunking	Memorization of new vocabulary by creating a story from the combination of mental images of vowel sounds with the meaning of the new word	Hermaphroditism could be chunked (Her-ma-phro-dit-is-m) and memorized as: Her mother from Detroit is an M&M – which is **M**ale and fe**M**ale
Visualization and storytelling	Creating (mental) images and stories to creatively connect new information (patterns) with existing long-term memory	P53 can be regarded as the guardian of DNA: imagine p53 as a person, a vigilant, heavily armed librarian who constantly monitors the library and reacts immediately to any signs of damage to books or media

In the realm of medicine, where intricate processes and numerous facts are the norm, mnemonics should prove useful.

#### Collaborative documents (CD)

Platforms such as Google Docs, Google Drive, Microsoft OneDrive, Wikis, and others could revolutionize the way we approach collaborative work and learning. The CDs on these platforms enable simultaneous input from multiple users and provide a space for creative interactions like document sharing, co-creating media, synchronous and asynchronous discussions, and modification of content in a fluid manner. This study opted for a device-independent, browser-based platform, in order to enable students to participate, regardless of their own devices. Therefore, we intentionally decided against solutions that would have required the installation of special software or applications.

The question arises whether CDs could have positive effects, such as improved collaboration, behavioral change, learning, or knowledge management [12]. There is some evidence that collaborative and peer-assisted learning can improve learning in the medical field [13, 14] In pathology, especially digital slides have been shown to improve student learning and collaboration [14–17]. Surprisingly, a PubMed search for ‘collaborative document’ in August 2023 yielded only 15 hits. None of these articles is related to medical education or pathology training. Important healthcare applications that use collaborative documents include clinical information systems, document repositories, or research environments [18–20].

In this paper, we present a pilot study of the combination of these two powerful tools, mnemonics, and CD.

We propose that peer creation and sharing of mnemonics in collaborative online platforms offers a dual benefit: the mnemonic aids in memory retention, while the collaborative nature of the platform allows for the refinement, discussion, creativity, and personalization of these mnemonics. This approach democratizes the process of creating and sharing mnemonics and fosters a sense of community and shared understanding among learners.

This study also contributes to the awareness of mnemonics and the possible uses of CD in medical education, in general.

## Methods

### Participants and setting

Third-year full-time students in human medicine were followed during the course of systemic histopathology. The intervention group (IG) was encouraged to generate creative mnemonics and share them on CD. The control group (CG) was asked to compare analog and digital microscopy. The curriculum included 10 weekly courses that lasted 60 min each. Thus, the time on task was identical for IG and CG. All courses were given by the author of this manuscript on Wednesdays when groups 3 and 4 were taught. Groups 1 and 2 were taught on Mondays and groups 5 and 6 Fridays by other lecturers. The first course was used for a pretest (T0), an introduction of the course, and a description of learning strategies, followed by 8 courses (cardiovascular system, respiratory system, gastrointestinal system, liver, lymph nodes, urinary system, genital system, mamma and skin), during which a total of 39 microscopic slides were taught. The digital slides of the course are freely available on the homepage of our university medical center, although individual slides or courses are subject to change [21]. The final course was used for the posttest (T1), evaluation, and general questions and answers.

Students were required to attend course sessions, but participation in the study was completely voluntary. There were no explicit assignments for students to prepare for courses or study for the tests. The pretest and posttest were administered only for this study and were not a mandatory requirement for obtaining the course certificate. To pass the official final exam of the course, students had to recognize and describe two out of three randomly assigned histopathological slides.

### Introduction to mnemonics

During the first course, students of the IG were introduced to mnemonics through a brief presentation. They also received a list of links to informative websites for self-directed learning of mnemonics (Table 2). Due to the limited available time, specific training of mnemonics could not be implemented during the courses.

**Table 2:** List of informative websites for self-directed learning of mnemonics

Contents	URL	Last access
General description of mnemonics and different mnemonic techniques:	https://de.wikipedia.org/wiki/Mnemotechnik [22]	May 18, 2024
Training opportunities for various mnemonics:	https://memocamp.com/de [23]	May 18, 2024
Information and training regarding mnemonics:	https://artofmemory.com/ [24]	May 18, 2024
Information, books, videos, courses for memory improvement by mnemonics:	https://www.nelsondellis.com/ [25]	May 18, 2024

### Introduction to collaborative documents (CD)

For each pathological case or histologic slide, a collaborative document was generated at https://drive.google.com/ [26]. A device-independent, browser-based platform was chosen so that students could participate regardless of their devices, without having to install special software or applications. A blank template can be found as an additional file [see Additional file 1]. The CD included four different sections.

- Section A: Mnemonics that connect the correct diagnosis with the macroscopic overview of the tissue on the slides, including outlines, shapes, or colors.
- Section B: Mnemonics that connect the terms of histopathologic highlights with familiar words that sound similar and make a memorable sentence or story.
- Section C: Mnemonics for other similarity of other new terms related to the specimen or the underlying disease.
- Section D: Mnemonics that would minimize the risk of confusion of slides with similar appearance or terms with a risk of confusion.

During the first course, students of the IG were also introduced to the benefits of peer creation and collaboration in CD. Students received live instructions on how to use the platform-independent browser-based CD. During the courses, the CDs were projected onto the screen and worked on while the respective microscopic slides were demonstrated by the lecturer and studied by the students. The co-creating process should lead to a greater number of mnemonics, inspiration from peers, supporting discussions, and also later asynchronous personalization and refinement of mnemonics. CD might even foster a sense of community among students. To overcome inhibition thresholds, all students could work completely anonymously. Diversity and equity could be considered appropriately by letting students choose an avatar of choice.

### Analog and digital microscopy

The students of the CG were free to choose digital slides with mobile devices or glass slides with analog microscopes. All digital slides of the course were freely available on the homepage of our university medical center 24/7 [21].

### Course design

All courses started traditionally for both groups. The teacher described histopathological slides using digital slides throughout the course. All cardinal features were demonstrated and explained and the students were allowed to ask questions.

CG students were asked to compare analog and digital microscopy. IG students received QR codes that led them to the CD, where they could simultaneously create and share mnemonics during and even after the course. All CDs were available to IG students throughout the semester.

### Pre- posttest design (quantitative data)

Participation in the study was voluntary. A pretest (T0) was performed before the first lesson and the interventions. The posttest (T1) was performed at the end of the semester. All tests were pseudonymized and included single choice and image recognition questions. In free text questions, students had to explain the pathogenesis and pathomechanisms or describe histopathological parameters.

### Interest, previous knowledge, and learning strategies (qualitative data)

The pretest before intervention (T0) and the posttest after intervention (T1) tests were supplemented with short questionnaires that were distributed to all students. Ratings were on a Likert scale from 1 (high / strongly agree) to 6 (low / strongly disagree).

The students were asked for:

- general interest in pathology
- their estimation of the importance of pathological learning objectives for their own future post-graduate medical training
- their previous knowledge of pathology
- their preference to study alone rather than collaboratively
- prior knowledge of mnemonics
- their preference for digital versus analog microscopy

At the end of the semester, open questions concerned:

- advantages and disadvantages of mnemonics (IG)
- advantages and disadvantages of collaborative documents (IG)
- advantages and disadvantages of analog or digital microscopy (CG)

The participants' responses were translated, structured, and paraphrased to capture the core meanings.

### Data analysis

The sample size was calculated with an online tool [27]. With an effect size of η^2^ = 0.1 (corresponding to an f of approximately.333, i.e., a small effect) and a power of 0.8, 37 subjects per group (74 total) were needed to obtain a significant result with a one-factor ANOVA (α = 0.05).

Quantitative data were analyzed by partial correlation, analysis of variance (ANOVA), multivariate analysis of variance (MANOVA), Shapiro–Wilk test for normal distribution, and Levene test for homogeneity of variances with SPSS 27 (IBM, Armonk, New York, USA).

Qualitative data were summarized by content and narrative analysis. Data from Likert scales were analyzed by the two-sample t-test assuming equal variances with SPSS 27 (IBM, Armonk, New York, USA).

## Results

The study was carried out according to the Declaration of Helsinki and was approved by the institutional ethics committee of the Rostock university hospital, reference number A 2019–0086.

### Paticipants and setting

Due to organizational reasons, a randomization was not possible. Therefore, we relied on a pseudo-experimental study design. The entire semester of about 240 students consisted of six study groups (1–6). Two groups were enrolled in the pilot study described here, because the author of this article only taught groups 3 and 4, whose courses took place consecutively on Wednesdays. Group 4 was randomly assigned the IG and group 3 the CG. In each group, 41 students voluntarily participated in the study. The sample size of 41 can be considered sufficient as the power calculation suggested a minimum of 37 students per group. In the CG, 78% of the participants were women (32/9) compared to 68% (28/13) in the IG (*p* = 0.4). There were no significant differences in age, which was 23.3 years in the IG and 22.9 in the CG (*p* = 0.6).

### The creation of mnemonics with CDs

The IG used CD for all 39 pathological cases / histopathological samples (100%). An abbreviated summary of all written entries of the students is provided as supplementary material [see Additional file 2]. Sometimes, students added screenshots of the digital slide overview (*n* = 16) to better recall the shapes and outlines of the tissue. Each of the 39 CD sections A (outlines, form, and color) contained between one and three mnemonics. Often, an analogous picture was used that resembled the shape or outline of the sample, occasionally including color. There were a total of 59 entries (1.5 entries / slide). In Section B, which had a total of 56 entries, 10 CD did not have entries related to histopathological highlights. An example in German is the pathological term ‘Ballen’, which refers to bales. The students created a mnemonic with a word that sounds similar: ‘Quallen’, which means jellyfish in English. Section C had only five entries, one of which was Bilharziose (English equivalent of Schistosomiasis), which students associated with ‘Bill Clinton’ stuck in 'Harz' (English equivalent of resin) with ‘aliens sprouting from his liver’, as the original histological slide showed the parasites in the liver. Section D had only one entry, which was pathological Langhans cells represented as pennies on a plate (for the distribution pattern of nuclei in multinucleated giant cells), not to be confused with physiological single nucleated dendritic LangERhans cells of the skin.

### Qualitative assessment of mnemonics and CD at the end of the semester

The open questions were summarized by content and narrative analysis. The IG was asked about the advantages of mnemonics and CDs. The most common answer was that the students could better remember the slides when they made associations of the shapes and outlines of the tissue with optical similarities. This simplified the learning process for 12 participants. Three learners each expressed their positive experiences with cooperation, collaboration, or finding it fun. Two students praised the effectiveness of mnemonics in improving their memory. Other students have reported being inspired by the innovation and have also found it useful that the CDs were available 24 h a day, 7 days a week.

As disadvantages, the students have mentioned that identification and documentation of mnemonics in collaborative documents takes additional time that should have been used for actual microscopic training (*n* = 5). Furthermore, the students mentioned that the mnemonics could distract from the actual pathological learning objectives (*n* = 5). Two students have reported experiencing technical difficulties with collaborative digital documents. They were unable to write or save documents on their mobile devices, especially smartphones. Other issues were inappropriate associations of other students that did not fit their own associations, lack of motivation, not enough pictures on the CD, not suitable for retrieval practice, and finally the statement ‘I don't use digital media for learning’.

### Quantitative evaluation of the pretest (T0) and posttest (T1) results

**Fig. 1:**
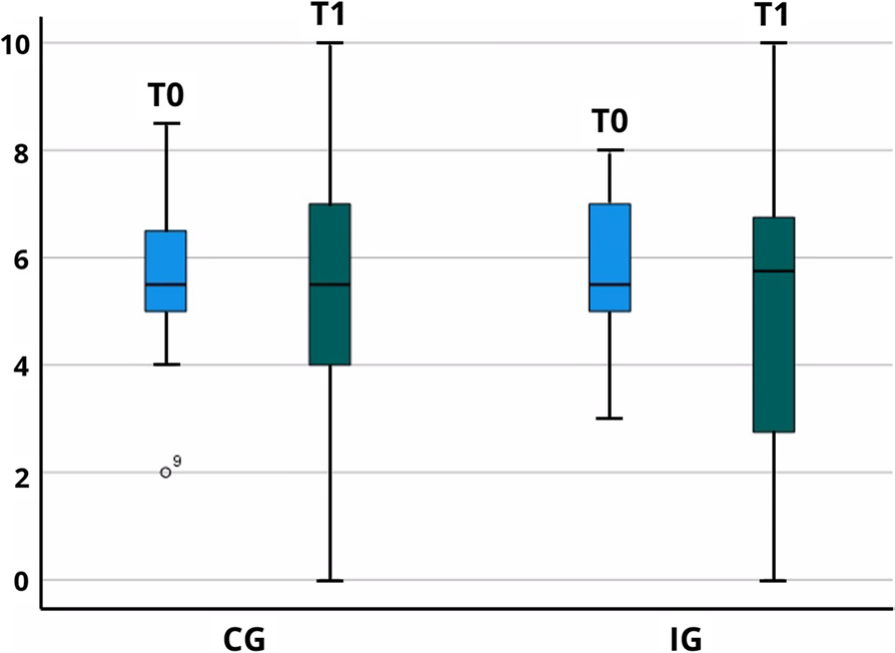
Box plot of IG and CG at T0 and T1

In the IG, 40 of 41 students completed the pretest T0 (98%), and 33 (80%) completed the posttest T1. In the CG, 36 of 41 students completed the pretest T0 (88%), and the posttest T1 by 34 of 41 (83%). No statistical differences were found for the test scores (Fig. 1).

The ANOVA in T0 revealed no significant differences in the test scores for IG (M = 5.7, SD = 1.4) and CG (M = 5.6; SD = 1.3) (F = 0.02; *p* = 0.9). The homogeneity of the variances was asserted using the Levene test, which showed that equal variances could be assumed at T0 (*p* = 0.5). ANOVA analysis in T1 also did not reveal significant differences in the test scores for IG (M = 5.1, SD = 2.6) and CG (M = 5.0; SD = 2.3) (F = 0.02; *p* = 0.9). Homogeneity of variances was asserted using the Levene test, which showed that equal variances could be assumed, also at T1 (*p* = 0.4).

In MANOVA analysis, the test scores for T0 and T1 were normally distributed for both groups, as assessed by the Shapiro–Wilk test (*p* > 0.05). There was homogeneity in error variances, as assessed by the Levene test (*p* > 0.05). The homogeneity of the covariance was verified using the box test (*p* = 0.844). Finally, there were no statistically significant differences between test scores and group, F (1.658) = 0.196, *p *= 0.66, partial η^2^ = 0.003.

### Qualitative assessment of interest, previous knowledge, and learning strategies

Our Likert scales ranged from 1 (high / strongly agree) to 6 (low / strongly disagree) and were analyzed using the two-sample t-test assuming equal variances. At T0, there were no significant differences in the means between the IG and the CG in terms of general interest in pathology (IG: 2.9; CG: 3; *p* = 0.5), the importance of pathological learning objectives for future postgraduate medical training (IG: 2.7; CG: 2.4; *p* = 0.2) or previous knowledge of pathology (IG: 3.7; CG: 3.7; *p* = 0.7).

In terms of learning strategies, IG and CG were not significantly different in their preference to study alone rather than collaboratively (IG: 3; CG: 2.4; *p* = 0.06), previous knowledge of mnemonics (IG: 3.2; CG: 3; *p* = 0.7) or in their preference for digital versus analog microscopy (IG: 3.2; CG: 3.4; *p* = 0.6).

### Qualitative assessment of analog and digital microscopy at the end of the semester

The open questions were summarized by content and narrative analysis. The most common response from CG to the advantages of digital microscopy was the availability 24/7 for self-directed learning (*n* = 17). This was followed by the quality and resolution of the digital slides (*n* = 8). Regarding the advantages of digital microscopy, two students appreciated its better usability for collaborative peer learning, the ability to identify structures with the cursor, easy handling, and the opportunity to learn using identical specimens, not parallel slides. Other comments included better orientation, students' ability to select their own slides, the opportunity to measure objects, zooming possibilities, annotations, and improved motivation.

The most common problems mentioned by the students were sometimes limited server availability and slow internet connections (*n* = 9), the quality and resolution of some slides (*n* = 3), differences between analog and digital slides, or loss of experience handling classical analog microscopes (two each). A single student reported that using digital slides was less fun.

## Discussion

Synthesizing peer creation and sharing of mnemonics in collaborative online documents in histopathological education represents an innovative teaching method that adapts to cognitive neurobiology and the digital transformation of medical education. In this pilot study, we investigated the feasibility and effectiveness of integrating the creation and sharing of mnemonics on CD platforms to improve pathology learning among third-year medical students. Our study design included qualitative and quantitative measures to capture the nuances of student experiences and learning outcomes.

### Mnemonics in pathology education

Whereas eponyms and acronyms, especially with food analogies, are an essential part of pathological textbooks, acrostics and other mnemonics are much rarer. A comprehensive literature search on mnemonics in pathology education revealed only 11 results [1–11]. Five of these are in the field of dermatopathology [2, 5, 6, 8, 10]. Most of these articles simply list a variable number of mnemonics. One larger study centered on food analogies and visualizations, but did not include a control group [9]. No prospective, randomized, controlled studies could be identified. In summary, this current literature does not reflect evidence-based research approaches. No obvious clues can be drawn for potential benefits or limitations, when we focus solely on pathology education. If we leave pathology didactics, a prospective randomized trial could show significant effects of mnemonics in the field of physiology [28]. Here, students in the mnemonics group scored significantly better in a pre-posttest setting. Although, our study could not prove a significant gain in pathological competences, the students stated that visual mnemonics simplified their learning process and improved their memory and that it was fun creating mnemonics. In family medicine, students also rated mnemonics very favorably, and they have proven useful for the generation of differential diagnosis [29].

As a limitation, our students mentioned inappropriate associations of other students’ metaphors that did not fit their own associations. This is a well-known phenomenon that is even more pronounced in students with different sociocultural backgrounds, which could have totally different associations with food-related medical terms [1, 11].

Other limitations were lack of motivation and that learning mnemonics distracted them from learning pathological learning objectives. These statements make it clear that whenever a new technology is used, educators and students should know why they are using the technique and why it is appropriate to use it in that particular context [30]. Another study found that students who received specific instruction in metaphor comprehension strategies performed better than those who did not receive specific instruction and who even tended to adopt a violation strategy [31]. We were able to observe something similar with our students, especially since visual similarities of specimen outlines were the most popular mnemonic aid. This is actually an incorrect use of mnemonics because students do not learn pathological learning objectives, but only remember the slides by the outline and color of the specimens instead of the histopathological features. Our students obviously took the easy route and focused only on passing the final exam, during which they had to recognize three randomly assigned histopathological slides. This unintended shortcut should be considered in future studies, where we will try to counteract the preference of our medical students for superficial learning approaches [32].

### Collaborative documents

The students praised and found it fun using CD by creating and sharing mnemonics. By giving students the privilege of creating and witnessing the development of mnemonics in real time, the CD served as a means of facilitating this process. Although our own data remain inconclusive in terms of knowledge gain, there is good reason to continue to monitor and encourage collaborative learning [12, 13, 17].

Other students mentioned the benefits of 24/7 access to the CD. The inclusion of mnemonics on the CD was intended to facilitate learning by drawing connections between histological characteristics and common memorable cues. Central to this approach is the belief that collaboration leads to greater expertise and fosters motivation and creativity for the creation of mnemonics [13, 15].

As a limitation, students have reported technical difficulties with collaborative digital documents, especially when using smartphones. This suggests that not all of our participants can be considered digital natives [33].

### Digital vs. analog microscopy

Digital microscopy has its advantages: availability, high resolution, and potential for collaborative peer learning [14–17]. However, challenges such as slow Internet connections and server problems cannot be ignored. As evidenced by our student feedback, there is a distinct nostalgia among other students for analog methods and the tangible experience they provide. Integrating digital and analog microscopy methods into future curricula could provide a more holistic learning experience.

### Limitations

The pilot study revealed three main limitations.

1. Wrong focus for mnemonics and not enough time for mnemonic creation, leading to a low number of mnemonics in CD.
2. Uniform test scores in the pre- and posttests.
3. Technical difficulties with the Internet and CD.

#### Too few mnemonics

Above, we already described the wrong focus of the students, using mnemonics mainly in section A only to memorize the specimen outlines and color, instead of the histopathological features. In sections B – C, only a few mnemonics were documented. The major problem was that it was too time-consuming to create useful mnemonics. Most of the students had only limited experience with mnemonics and, as with every new method, there is a variable learning curve that makes the creation of new mnemonics challenging for certain terms or concepts. Some students found the process to be not only time consuming, but also distracting. These findings suggest that while the integration of mnemonics in CDs has potential in pathology education [2, 9, 11], the time management, the process, and a more meticulous design of the instructions need to be refined. A more meticulous educational design [34, 35] could prove more effectively, against our students for superficial learning approaches [32].

#### Uniform test scores

Despite the introduction of this promising and novel cognitive neurobiological method, no significant differences were observed in the pretest and posttest scores between the IG and the CG. Although all students received the same set of questions, we restricted the test questions to a minimum and did not validate them. Even more importantly, our pre- and posttests were not a prerequisite for passing the course and getting all credits. The tests were only conducted for the purpose of this study. Although exams are one of the main motivators to learn, they lose their effect when all theories of adult learning are bypassed if the exams are not relevant to passing [36]. In our cohort, the mediocre performance of all students in the pre- and posttest ultimately suggests that the two tests were too difficult for the students and were also not taken seriously. A follow-up study would have to use validated tests, which would also be decisive for passing the course.

#### Technical difficulties

Lack of accessibility or other problems with digital tools can deter participation and compromise learning outcomes. The students identified two different technical problems. One was related to digital microscopy and the other to CD. Regarding digital microscopy, our old platform in 2019 was obviously insufficient. It was a locally administered server with password protection. Meanwhile, all data are barrier-free on high-performance servers, allowing freely, reliably, easily, and fast access [21]. Regarding CD, we thought that we would already work with 'digital native students'. However, it became clear that the students had little or no experience using CDs with peers. Furthermore, some students stated that not all mobile devices, especially smartphones, managed CD with no difficulties. Therefore, we conclude that only a few, if any, ‘digital natives’ were enrolled in the 2019 course [33].

## Implications for future studies

Various interpretations and potential improvements arise from our data.

### Clear instructions

To establish a new learning technique and implement new digital learning tools, care must be taken to provide adequate explanations and follow instructional design rules for mnemonics and for CD [34, 35].

### Counteracting students’ bypasses

It must be taken into account that mnemonics focus on real pathological learning objectives and not support the preference of medical students for superficial learning approaches [32].

### Enhanced training

Future implementations should consider more comprehensive training for students in the creation and sharing of effective mnemonics. The faster students develop mnemonics, the more time they will have for practical pathological training. An online inverted classroom model might help to learn how to create more functional mnemonics [37].

### Mnemonic Individuality and quality

The collaborative nature, while beneficial for brainstorming, could become a source of confusion for some students. The challenge in a collaborative setting is to ensure mnemonics that resonate with the majority or to have enough mnemonics so that students can choose one that works best for them. However, the efficacy of a mnemonic is largely dependent on its relevance and ease of understanding. If the mnemonics created were not sufficiently relevant or memorable, their utility would be limited [2]. Our study did not assess the quality or relevance of the generated mnemonics. Future studies could include a qualitative analysis of mnemonics to assess their relevance and potential impact on learning outcomes.

### Cognitive load

The process of developing mnemonics could create an additional cognitive load [38], potentially distracting from the core histopathological content. This is consistent with student feedback, where some found the mnemonics distracting. Students are required to acquire a vast amount of knowledge and skills, and a deliberate approach to instructional design is needed to provide students with effective learning strategies [34, 35].

### Time on task

As the students noted, the extra time spent on identification and documentation of mnemonics was removed from hands-on microscopy time. Obviously, there is a learning curve associated with adopting new study methods like creating mnemonics and working collaboratively on CD. Students may need more time and guidance to effectively integrate this technique into their study habits, which could dilute short-term results but prove beneficial in the long run.

### Validated high-stakes testing

Most educators agree that (especially standardized) assessment drives learning [39]. For quality assurance, the tests should be validated and standardized in advance. Furthermore, the test should be mandatory to receive full credit for the course [36].

### Technical difficulties

All digital tools, be they digital microscopy or CD, must be barrier-free (no passwords, free of charge), robust, user-friendly, and compatible with multiple devices, that is, platform-independent and browser-based.

### Train the trainer

Pathologists as long-term teachers could be trained in a pathological didactic curriculum to better address the needs of their students and keep up with new evidence-based teaching methods [40].

### Larger sample size

Although the sample size in this study was statistically appropriate for a pilot study or a feasibility study, a larger cohort could provide more nuanced information.

## Conclusions

This pilot study provides a foundation on which future research can be built. Although the creation and sharing of mnemonics in CD offers promising opportunities to improve memory in pathology education, it is clear that the approach needs optimization. Despite the evidence-based, prospective, quasi-experimental, pre- posttest, mixed methods approach, a greater focus is imperative on educational and instructional design [34, 35]. Dividing the sessions into mnemonic creation and practical histopathology could ensure focused learning. An online blended learning approach might also be beneficial [37]. Additionally, more reliable technological platforms are needed to evaluate digital slides, and working on CD may be the way forward. Although there is some evidence that student performance can be enhanced by desirable difficulty [38], we must be careful not to overburden our students. Finally, validated high-stakes tests should be used for our future pre-posttest study.

### Supplementary Information

Additional file 1. Blank template of a collaborative document, translated into English.
Additional file 2: Supplementary Table 3. Summary of translated student entries in CD; Description: Sections A and B appear in columns 2 and 3. The few entries in Sections C (new terms related to the specimen or the underlying disease reminded me of,…) and D (Risk of confusion with…) appear in additional inserted rows.

## Data Availability

No datasets were generated or analysed during the current study.

## Abbreviations

ANOVA: Analysis of variance
CD: Collaborative documents
CG: Control group
IBM: International business machines
i.e.: Id est: Latin for ‘in other words’
IG: Intervention group
MANOVA: Multivariate analysis of variance
SPSS: Statistical package for social sciences
T0: Pretest
T1: Posttest
USA: United States of America
