# Supplementary material for: Peer creation and sharing of mnemonics in collaborative documents for pathology education: a pilot study
Source: BMC Med Educ. 2024 Jul 9;24:742. doi: 10.1186/s12909-024-05743-1 (PMC11234616; doi:10.1186/s12909-024-05743-1)
Supplement: Supplementary file 1 — Additional file 1. Blank template of a collaborative document, translated into English. Additional file 2: Supplementary Table 3. Summary of translated student entries in CD; Description: Sections A and B appear in columns 2 and 3. The few entries in Sections C (new terms related to the specimen or the underlying disease reminded me of,…) and D (Risk of confusion with…) appear in additional inserted rows. [file 12909_2024_5743_MOESM1_ESM.pdf]

Universität Rostock — Traditio et Innovatio

Universitätsmedizin Rostock

Course Systemic Pathology # (1 - 8)

Specimen: ________________________

**A: Macroscopic overview of the tissue on the slides, including outlines, shapes, or colors of tissue reminds me of:**

**B: Histopathological highlights**	Terms remind me of:

**C: Other similarity or new terms**	… remind me of:

**D: Mnemonics that would minimize the risk of confusion of slides with similar appearance or terms with a risk of confusion:**

Thank you for your contribution.
